# Supplementary material for: Stillage Waste from Strawberry Spirit Production as a Source of Bioactive Compounds with Antioxidant and Antiproliferative Potential
Source: Antioxidants (Basel). 2023 Feb 9;12(2):421. doi: 10.3390/antiox12020421 (PMC9951990; doi:10.3390/antiox12020421)
Supplement: Supplementary file 1 [file antioxidants-12-00421-s001.zip › antioxidants-2206634-supplementary.pdf]

Stillage Waste from Strawberry Spirit Production as a Source of Bioactive Compounds with Antioxidant and Antiproliferative Potential

Carmela Spagnuolo [1,*], Federica Moccia [2], Idolo Tedesco [1], Eva Adabbo [1], Lucia Panzella [2,*], Gian Luigi Russo [1] and Alessandra Napolitano [2]

[1] Institute of Food Sciences, National Research Council, I-83100 Avellino, Italy
[2] Department of Chemical Sciences, University of Naples "Federico II", I-80126 Napoli, Italy
* Correspondence: carmela.spagnuolo@isa.cnr.it (C.S.); panzella@unina.it (L.P.); Tel.: +39-0825299261 (C.S.); +39-081674131 (L.P.)

Table of contents **Page**

Figure S1. HPLC profiles of SE. 1

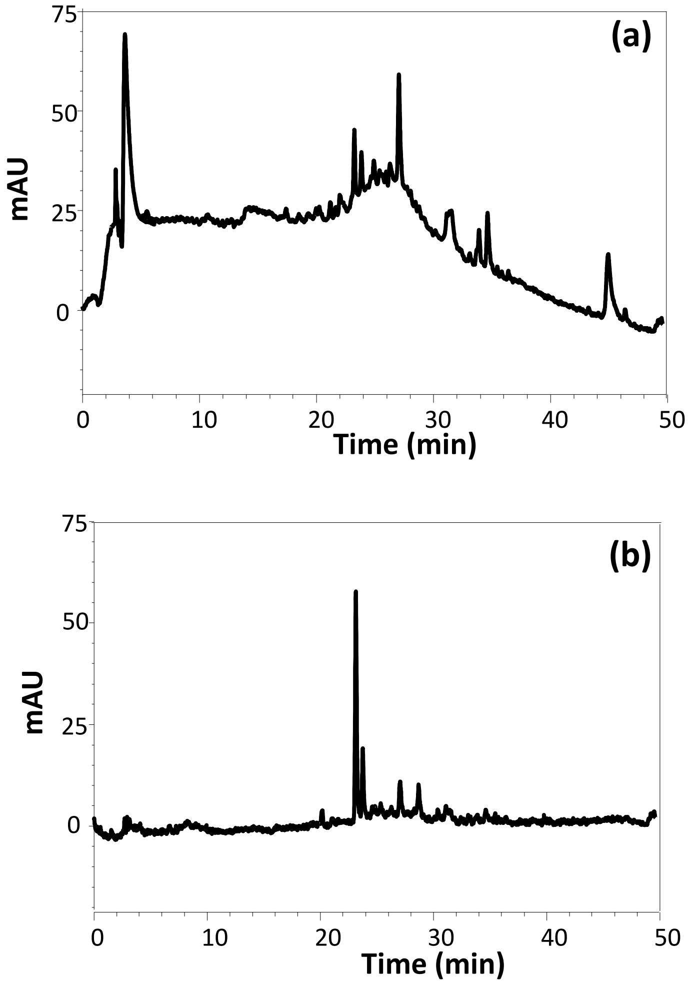

Figure S1. HPLC profile of SE (diluted 1:5 v/v in methanol), recorded at (a) 254 nm and (b) 320 nm.
